# 

**Published:** 1867-07

**Authors:** 


### THE CHICAGO MEDICAL JOURNAL.

Edited by J. ADAMS ALLEN, M.D., LL.D.

Prof. Principles and Practice of Medicine and Clinical Medicine in Rush Med. College.

All letters for the JOURNAL should be addressed to DR. J. ADAMS ALLEN, P.O. Box 1948, Chicago, Ill.

**TERMS---$2 PER ANNUM, IN ADVANCE.**

$3 if not paid before 1st of July in each year. Single copies, 25 cents.

